# Follicular Dowling-Degos Disease Camouflaged as Comedones: A Case Report and Literature Review

**DOI:** 10.7759/cureus.26078

**Published:** 2022-06-19

**Authors:** Arundhathi S, Poongodi Rajagopal, Hima Gopinath, Jami Rupa Ramani

**Affiliations:** 1 Pathology, All India Institute of Medical Sciences, Mangalagiri, Guntur, IND; 2 Dermatology, All India Institute of Medical Sciences, Mangalagiri, Guntur, IND

**Keywords:** flexural hyperpigmentation, pitted scar, genodermatosis, reticulate hyperpigmentation, antler horn-like, comedones, dowling-degos disease

## Abstract

Dowling-Degos disease (DDD) is an uncommon autosomal dominant genodermatosis that resides in the spectrum of diseases presenting with reticulate pigmentation. This disease has varied phenotypic expressions, the classical presentation being reticular pigmentation of flexures involving the axilla, submammary folds, inguinal folds, and neck. Follicular DDD is a variant of DDD with a unique presentation of folliculocentric papules, macules, pits, and comedones associated with the characteristic histological findings of follicle-centered, pigmented, branching, antler horn-like rete ridges sparing the interfollicular epidermis. Due to the rarity and paucity of data about this entity, we describe this case of a 28-year-old female who presented with perioral pitted scars and multiple hyperpigmented folliculocentric comedo-like papules over the face, neck, cubital fossa, and upper trunk, unaccompanied by the typical non-follicular, reticulate flexural hyperpigmentation, which clinically posed a diagnostic challenge. The diagnosis was confirmed by histopathology. We intend to increase clinicians’ cognizance with respect to the unique clinical and histopathologic presentation of follicular DDD. More genetic studies could bring more understanding of this complex spectrum.

## Introduction

Dowling-Degos disease (DDD) was described by Wilson Jones and Grice in 1978 [1]. It is a rare genodermatosis usually with an autosomal dominant mode of inheritance but can also be sporadic [2]. Mutations in the keratin 5 (KRT5) gene located on chromosome 12q are responsible for the occurrence of DDD. The dysfunctional keratin gene leads to defective sebaceous glands and hair follicular structure. The typical clinical presentation is reticulate hyperpigmentation involving flexures of the axilla, submammary folds, inguinal folds, and neck.

Follicular DDD is a variant of DDD introduced by Singh et al. in 2013 [3]. This variant of DDD is reported to have a unique clinical presentation as punctate folliculocentric hyperkeratotic hyperpigmented papules, macules, pits, and comedo-like lesions mainly involving the face, back, extremities, and flexures with histological changes limited to follicular infundibulum sparing the interfollicular epidermis. The usual presentation of the non-follicular type of hyperpigmentation of flexures seen in classical DDD is characteristically absent in follicular DDD [3]. To the best of our knowledge, only 13 cases (including our case) have been reported in the English literature till now [3-9]. Hence, we write this case to highlight the typical clinical and histopathologic presentation of follicular DDD.

## Case presentation

**Figure 1:**
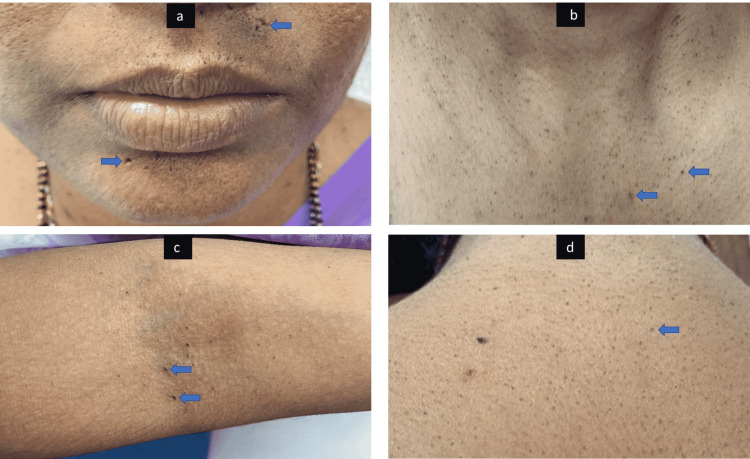
Photograph of the patient showing (A) multiple perioral pitted scars (two of them are highlighted by arrows). (B) Multiple folliculocentric comedo-like papules in the neck (two of them are highlighted by arrows). (C) Multiple comedones in the cubital fossa (two of them are highlighted by arrows) and the absence of typical flexural hyperpigmentation. (d) Multiple folliculocentric comedo-like papules in the upper back (one of them is highlighted by an arrow).

**Figure 2:**
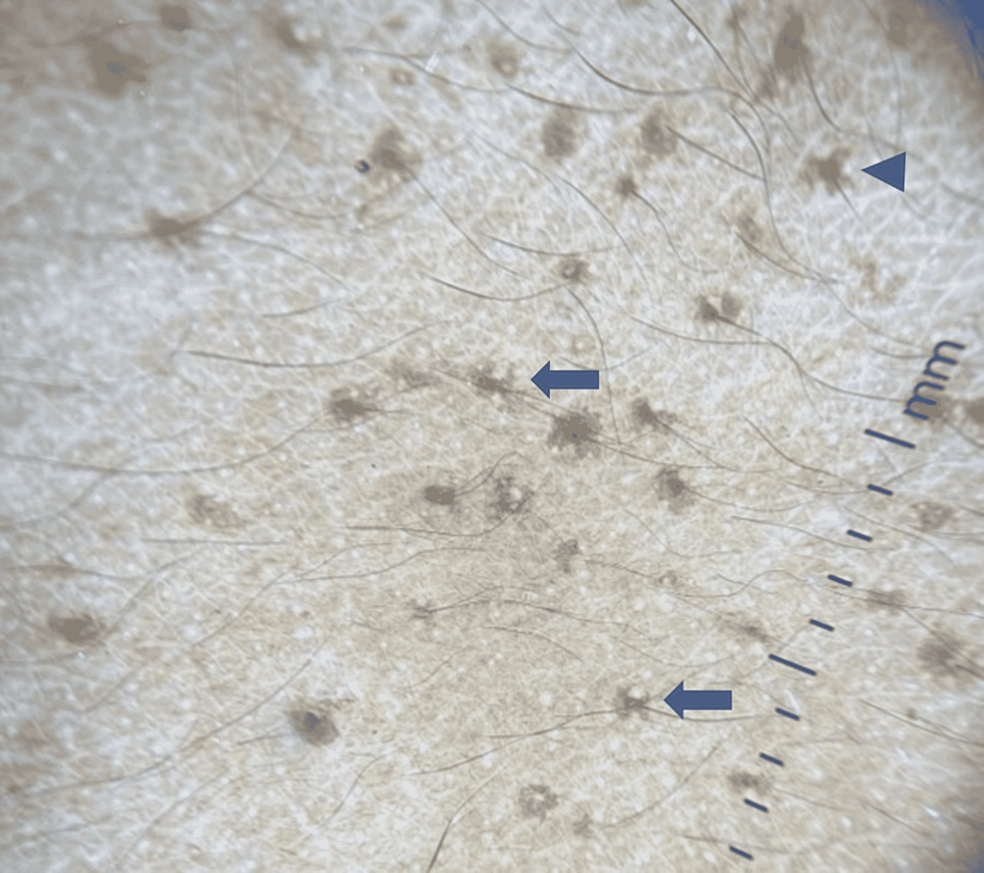
Dermoscopy in polarized mode (10x magnification) of the upper back showing folliculocentric irregular Chinese letter-like (arrows) and star-like (arrowhead) brown pattern.

A 28-year-old female presented with perioral pitted scars, comedones over the cubital fossa, and multiple hyperpigmented folliculocentric comedo-like papules over the face, neck, and upper trunk (Figure 1) for five years and denied any similar lesions in other family members. Reticulate hyperpigmentation in flexures was not present. Dermoscopy in polarized mode (10x magnification) of the upper back revealed folliculocentric irregular star-like and Chinese letter-like brown pattern (Figure 2). The differential diagnoses considered were familial dyskeratotic comedones, familial comedonal Darier's disease, and DDD.

**Figure 3:**
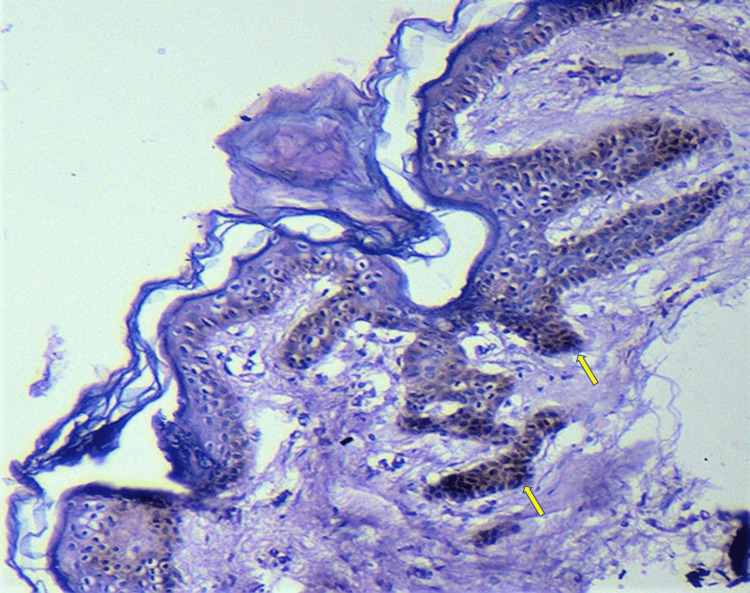
Microphotograph showing follicular plugging with antler horn-like elongations of rete pegs emanating from follicular infundibulum with melanin concentration over the tips of rete (arrows) (hematoxylin and eosin, 100x).

Histopathological examination from the comedo-like papule revealed hyperkeratotic epidermis follicular plugging along with antler-like elongation of rete ridges confined to follicular infundibulum with melanin concentrated over the tips of rete (Figure 3). The interfollicular epidermis was uninvolved. No dyskeratotic cells or corps ronds or corps grains were seen. These features were consistent with the diagnosis of follicular DDD. Genetic analysis could not be done. The patient was started on topical 0.1% adapalene gel. There was a mild improvement noted on follow-up.

## Discussion

DDD is a rare hyperpigmentation disorder. It occurs due to mutation in the KRT5 gene as an autosomal dominant genodermatosis or sporadically. It shows a female predilection presenting in the third or fourth decade [2]. Our index case is also a young female. However, she denied any similar lesions in other family members. The reason behind that may be the patient is incognizant or it can be a sporadic occurrence. Also, the family members were not available during her visit for physical examination. Genetic studies are not carried out in this case due to affordability issues and the classical histopathology features clinched the diagnosis.

The causative mutation in the KRT5 gene located on the long arm of chromosome 12 is known to cause abnormal epithelial proliferation of the pilosebaceous unit and thereby the occurrence of DDD. KRT5 gene is involved in cell adhesion and the transfer of melanosomes [2]. DDD is also known to be associated with other mutations in POFUT1 (O-fucosyltransferase 1) and POGLUT1 (O-glucosyltransferase 1) [2]. It has a delayed presentation around the third to the fourth decade of life. It presents as reticular pigmentation in intertriginous areas like the axilla, groin, inframammary folds, and axilla. As the disease progresses with time, it involves other unusual sites like the trunk, inner thighs, upper arms, and face [2].

Other findings in DDD include hypopigmented macules and papules, comedones-like lesions, fingernail dystrophy, pitted perioral scars, chloracne-like presentation, Galli-Galli disease, and overlap with reticulate acropigmentation of Kitamura (RAPK) [10-12]. Many reports have also revealed the association of DDD with epidermal cysts, hidradenitis suppurativa, keratoacanthoma, and perianal squamous cell carcinoma [2,6,13]. Henceforth, DDD is characterized by varied phenotypic expression with a reticulate pattern of flexural hyperpigmentation being the commonest presentation.

Follicular DDD is a variant of DDD, first described by Singh et al. [3] in 2013. They reported two cases with positive family history: one patient had multiple follicular macules, pits, and few keratotic follicular papules over the face, upper back, extremities, ears, bilateral axillae, popliteal, and cubital fossae, and the other patient revealed pitted scars over the face and upper back, and comedo-like hyperpigmented follicular papules over the nape of the neck and back along with few inflammatory nodules over the upper back. No flexural accentuation of the lesion was noted in their study. They identified in the biopsy that the morphological changes are limited to follicular infundibulum sparing the interfollicular epidermis and diagnosed them as follicular DDD [3].

Following their study, few of the authors have published and documented the same type of clinical presentation and histological changes in follicular DDD supporting and adding more value to their findings [4-9].

Recently, dermoscopy of DDD studied revealed irregular star-shaped or linear thready pigmentation in Chinese letter pattern seen over follicular papules as a suggestive feature of follicular DDD. Thus, dermoscopy is being used as a diagnostic tool to diagnose follicular DDD [5]. The clinical diagnosis is easy when the typical features of the disease and dermoscopy are present. In cases lacking classical features, histopathology plays a pivotal role.

DDD is histologically characterized by irregular elongation of thin branching rete ridges with increased melanization at the tips giving an “antler-like” pattern. Follicular DDD shows similar histological changes only around the follicular infundibulum with follicular plugging and horn cyst formation sparing the interfollicular epidermis [3].

The differential diagnostic possibilities of DDD include Galli-Galli disease, familial dyskeratotic comedones, and familial comedonal Darier’s disease. Galli-Galli disease is a variant of DDD characterized by non-dyskeratotic acantholysis [2]. The presence of typical Darier's nails and palmar features with nodular keratotic lesions on the face, upper trunk, and scalp with corps ronds and grains on histology are the features of comedonal Darier's disease [14]. Familial dyskeratotic comedones present as multiple, open comedones mainly in the pubertal age group and they involve arms, trunk, legs, sparing palms, and soles; a biopsy of the lesion will show dyskeratosis and dermal invagination with occasional acantholysis [15]. In our case, the characteristic acanthotic projections with follicular infundibular involvement without any associated acantholytic cells, dyskeratotic cells, corps ronds, and corps grains helped us to exclude the above differentials.

There are other disorders with overlapping clinical and histopathological features of DDD. To categorize them, these disorders were divided into two major groups. The first is classic DDD with typical histologic characters and the generalized DDD with generalized hypopigmented papules [16]. The second group consisted of dyschromatosis like dyschromatosis symmetrica hereditaria (DSH), dyschromatosis universalis hereditaria (DUH), and RAPK. Thus, reticulate pigmentary disorders are a gamut of diseases with overlapping clinical pictures and histopathology will ease in differentiating these entities [12].

Treatment options such as retinoic acid, hydroquinone, erbium-YAG (yttrium aluminum garnet), and a combination of Q-switched Nd:YAG and fractional carbon dioxide laser have been tried with limited benefits [17].

## Conclusions

DDD can have varied presentations and remains camouflaged. In our case of follicular DDD, there is an absence of the usual presentation of non-follicular reticulate flexural hyperpigmentation clinically and the case primarily presented as folliculocentric comedo-like papules with atrophic scars and in dermoscopy as folliculocentric pigmentation and in biopsy as a follicle confined lesion with typical acanthotic filiform projections. Clinicians’ cognizance of this unique presentation is essential for correct diagnosis and management. Genetic analysis is further needed to better understand the complex spectrum.
